# A spike virosome vaccine induces pan-sarbecovirus antibody responses in mice

**DOI:** 10.1016/j.isci.2024.109719

**Published:** 2024-04-10

**Authors:** Mitch Brinkkemper, Meliawati Poniman, Esther Siteur-van Rijnstra, Widad Ait Iddouch, Tom P.L. Bijl, Denise Guerra, Khadija Tejjani, Marloes Grobben, Farien Bhoelan, Denzel Bemelman, Ronald Kempers, Marit J. van Gils, Kwinten Sliepen, Toon Stegmann, Yme U. van der Velden, Rogier W. Sanders

**Affiliations:** 1 Amsterdam UMC, location University of Amsterdam, Department of Medical Microbiology and Infection Prevention, Meibergdreef 9, 1105 AZ Amsterdam, the Netherlands; 2 Amsterdam institute for Infection and Immunity, Infectious diseases, Meibergdreef 9, 1105 AZ Amsterdam, the Netherlands; 3 Amsterdam UMC, location University of Amsterdam, Department of Experimental Immunology, Meibergdreef 9, 1105 AZ Amsterdam, the Netherlands; 4 Mymetics BV, JH Oortweg 21, CH 2333 Leiden, the Netherlands; 5 Department of Microbiology and Immunology, Weill Medical College of Cornell University, New York, NY, USA

**Keywords:** Immunology, Virology

## Abstract

Zoonotic events by sarbecoviruses have sparked an epidemic (severe acute respiratory syndrome coronavirus [SARS-CoV]) and a pandemic (SARS-CoV-2) in the past two decades. The continued risk of spillovers from animals to humans is an ongoing threat to global health and a pan-sarbecovirus vaccine would be an important contribution to pandemic preparedness. Here, we describe multivalent virosome-based vaccines that present stabilized spike proteins from four sarbecovirus strains, one from each clade. A cocktail of four monovalent virosomes or a mosaic virosome preparation induced broad sarbecovirus binding and neutralizing antibody responses in mice. Pre-existing immunity against SARS-CoV-2 and extending the intervals between immunizations enhanced antibody responses. These results should inform the development of a pan-sarbecovirus vaccine, as part of our efforts to prepare for and/or avoid a next pandemic.

## Highlights

• Influenza virosomes can co-display diverse sarbecovirus spike proteins
• Multivalent sarbecovirus spike vaccine induces broad humoral immune responses
• Pre-existing SARS-CoV-2 immunity improves immune responses after experimental vaccinations
• Extended intervals between vaccinations improve vaccine induced immune responses

## Graphical abstract

**Figure:**
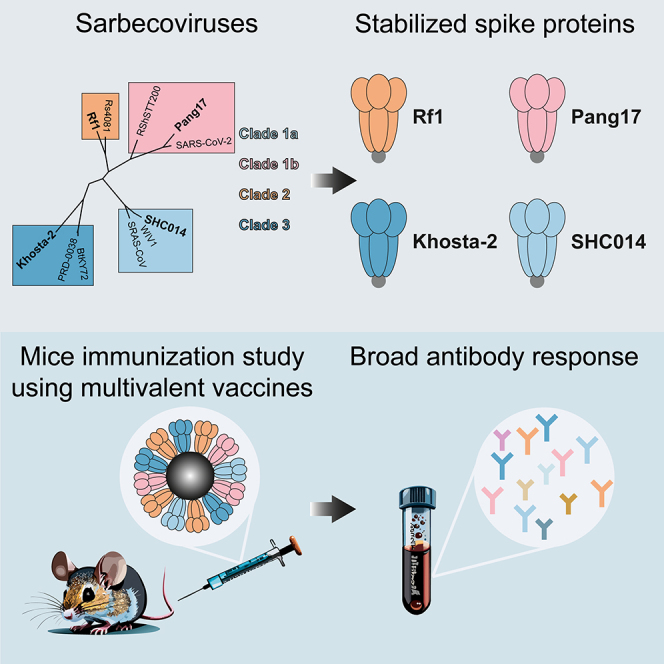

## Introduction

The severe acute respiratory syndrome coronavirus (SARS-CoV) and SARS-CoV-2 sarbecoviruses have caused an epidemic and a pandemic, respectively, in the 21st century. The SARS-CoV outbreak in 2002–2003 resulted in ∼8,000 probable cases and ∼900 SARS-related deaths.^1^ Since its first detection in 2019, SARS-CoV-2 has caused over 750 million infections and close to 7 million confirmed SARS-CoV-2-related deaths (https://covid19.who.int/). Both viruses are of zoonotic origin, with bats acting as suspected reservoirs.^2^^,^^3^ Sarbecoviruses are divided into four different clades: 1a, 1b, 2, and 3. SARS-CoV and SARS-CoV-2 belong to clades 1a and 1b, respectively. Sarbecoviruses from all clades have been found in animals.^4^ Diverse SARS-related coronaviruses (SARSr-CoVs) have been detected in bats, but also in other species, including civets and pangolins.^5^^,^^6^^,^^7^ A serological surveillance detected anti-SARSr-CoV reactivity in people who live in close proximity to caves in which bats carrying SARSr-CoVs roost, suggesting that several bat SARSr-CoVs are able to directly infect humans.^8^ Such viruses pose an ongoing threat and, therefore, a pan-sarbecovirus vaccine that could provide protection against circulating sarbecoviruses would be a desirable tool for pandemic preparedness efforts.

Neutralizing antibodies (NAbs) are one of the main correlates of protection against SARS-CoV-2 infection and coronavirus disease 2019 (COVID-19).^9^^,^^10^^,^^11^ The target for NAbs against SARS-CoV-2 and other sarbecoviruses is the spike glycoprotein (S). S is a class 1 viral fusion protein that interacts with a host cell receptor to enable merging of the viral and host cell membranes.^12^ The receptor used by sarbecoviruses from clades 1 and 3 is angiotensin-converting enzyme 2 (ACE2),^13^ while the receptor for clade 2 viruses is currently unknown. S is a trimer of heterodimers which consists of the transmembrane subunit S1, and the receptor-binding subunit S2. To initiate membrane fusion, the S1 subunit is proteolytically released, and S2 undergoes a number of large conformational changes to bridge the cellular and viral membranes.

The main target for NAbs is the receptor binding domain (RBD), and RBD-only vaccines have been shown to be effective. The immunogenicity of RBD can be improved by multimerization, and candidate pan-sarbecovirus vaccines using diverse multimerized RBDs have been described.^14^^,^^15^^,^^16^ However, most SARS-CoV-2 vaccines currently in use employ the complete ectodomain of S, often stabilized by the addition of prolines to generate stabilized prefusion S trimers.^17^ Prefusion stabilized S usually induces stronger Ab responses compared to RBD.^18^^,^^19^ NAb epitopes other than RBD, including the N-terminal domain (NTD) in S1 and epitopes in S2, might contribute to the induction of cross-reactive antibodies. Here, we exploit multimerized prefusion stabilized S based on four SARSr-CoVs, one from each sarbecovirus clade, for a pan-sarbecovirus vaccine.

Multimeric antigen presentation is a well-established strategy for inducing strong humoral immune responses. Multiple immunological processes are aided by multivalent presentation of antigens on nanoparticles (NPs). These include retention on follicular dendritic cells, lymph node trafficking, and strong B cell activation.^20^^,^^21^ In this study, we deployed an influenza virosome platform.^22^^,^^23^ Several virosome-based vaccines have previously been licensed.^24^^,^^25^ The virosomes used here constitute the reconstituted membranes of influenza virus containing the hemagglutinin (HA) and neuraminidase (NA) glycoproteins. The influenza components can aid the immune response through intrastructural help.^26^^,^^27^ The particles measure ∼100 nm and are modified to display any antigen of choice. We have previously shown that virosomes displaying SARS-CoV-2 S induced potent NAb responses against SARS-CoV-2 variants in mice.^28^ The ability of virosomes to induce strong immune responses can be enhanced by physically incorporating adjuvants, including the TLR7/8-agonist 3M052,^28^ as well as QS-21.

Here, we assessed in mice the immunogenicity of monovalent sarbecovirus S-virosomes, each containing S based on one virus strain from each sarbecovirus clade, a mosaic virosome displaying all four S proteins on its surface, or a cocktail of the four monovalent virosomes. To mimic a coronavirus-experienced immune system, we also assessed the consequences of pre-vaccination with SARS-CoV-2 S and S of the human common cold virus OC43. Finally, we investigated whether extending the interval between immunizations affected the strength of the immune response.

## Results

### Animal sarbecovirus S trimers are recognized by Abs isolated from human SARS and COVID-19 survivors

**Figure 1:**
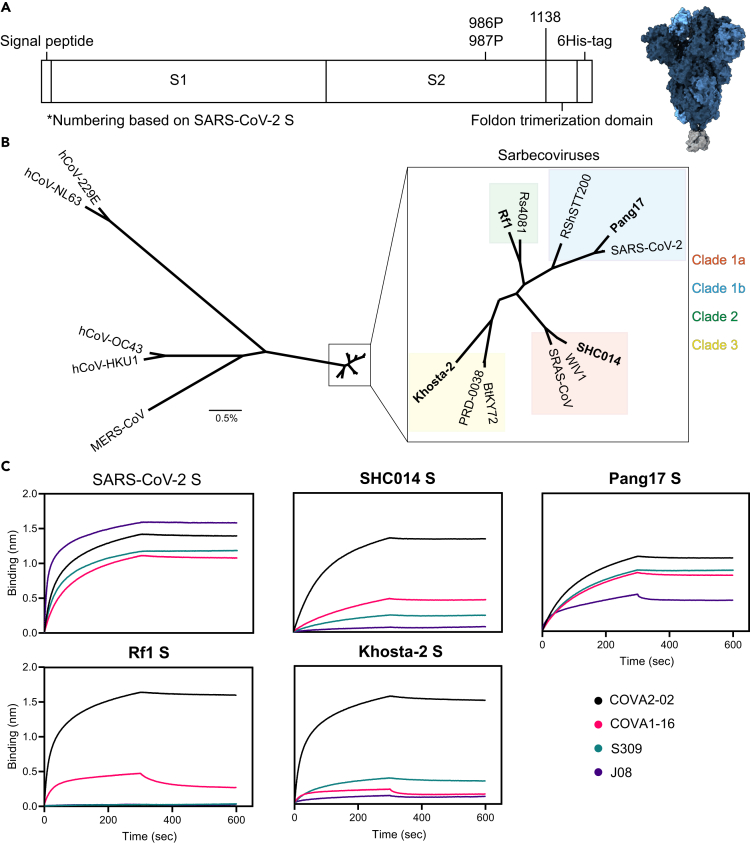
Sarbecovirus S production (A) Linear schematic of the prefusion stabilized S constructs (left), and 3D structure (right). The different protomers of S are shown in different shades of blue (PDB: 6VXX). The foldon trimerization domain is shown in gray (PDB: 4NCU). Numbering based on SARS-CoV-2 S. (B) A phylogenetic tree showing variation between hCoVs, MERS-CoV and sarbecovirus S proteins studied here. The horizontal bar indicated 0.5% variation. The sarbecovirus clades are indicated by different colors, and the strains used in the immunization study are in bold. (C) BLI sensorgrams of binding reactions of monoclonal Abs isolated from SARS-CoV-2 or SARS-CoV survivors to the four sarbecovirus S proteins used in immunization study, compared to SARS-CoV-2 S. See also Figures S1 and S2.

We generated stabilized prefusion S proteins derived from 11 sarbecoviruses from clades 1a, 1b, 2, and 3 (Figure 1). To stabilize S proteins in the prefusion conformation, prolines were introduced at positions 986 and 987, the transmembrane domain was truncated at position 1,138, and a C-terminal foldon trimerization domain was added (numbering based on SARS-CoV-2 S). In SARS-CoV-2 S, a GGGG substitution at position 682–685 was used to remove the furin cleavage site, while the other S proteins naturally lack such a site. A C-terminal hexavalent His-tag was used for purification and coupling to virosomes (Figure 1A). At least 2 S proteins of each sarbecovirus clade were selected for production: SARS-CoV, SHC014, and WIV1 from clade 1a; SARS-CoV-2, Pang17, and RshSTT200 from clade 1b; Rf1 and Rs4081 from clade 2; BtKY72, Khosta-2, and PRD-0038 from clade 3 (Figure 1B), of which one of each clade was selected for the immunization study based on which S proteins produced the best: SHC014, Pang17, Rf1, and Khosta-2. S proteins were produced in HEK293F cells and purified as previously described.^29^ Blue native PAGE analysis revealed that the samples contained mostly trimeric S and some aggregates, and in some samples we observed evidence of trimer degradation (Figure S1A). A bio-layer interferometry (BLI)-based assay using cross-reactive Abs was performed to assess the antigen conformation. These Abs included COVA2-02, COVA1-16, and J08 isolated from SARS-CoV-2 infected individuals, and S309, derived from a SARS survivor in 2003.^30^^,^^31^^,^^32^ All four Abs target the RBD, and all neutralize SARS-CoV-2, while COVA1-16 and S309 also neutralize SARS-CoV.^30^^,^^32^ All sarbecovirus S constructs bound strongly to COVA2-02. The S proteins from SHC014, WIV1, Pang17, RshSTT200, Rf1, Rs4081, BtKY72, and PRD-0038 interacted with COVA1-16, but binding of Khosta-2 S to this Ab was weak. Pang17 S showed strong binding to S309, while SHC014 and Khosta-2 S showed weak binding, and Rf1 S did not bind to S309. Finally, Pang17 S interacted efficiently with J08, while no or very low binding to J08 was observed with SHC014, Rf1, and Khosta-2 S (Figures 1C and S1B). Given that all S proteins bound efficiently to at least one and often multiple cross-reactive RBD Abs we infer that they present immune-relevant conformations.

### Multivalent S-virosomes induce potent antibody responses in mice

The SHC014, Pang17, Rf1, and Khosta-2 S proteins were coupled to detergent-solubilized influenza virus membranes that were around 100 nm in size, and contained lipid head-groups modified for click-chemistry (DBCO-PE) (Figure S2). A 3-fold excess of DBCO was available on the virosomal lipids for coupling to the azide on S. Proteins were conjugated to the virosomes via azide-DBCO click chemistry.^28^ For mosaic preparations, the S proteins were mixed at equimolar amounts before conjugation. All virosome vaccinations were adjuvanted by incorporating 3M-052 and QS21 into the virosomes.^28^^,^^33^^,^^34^

**Figure 2:**
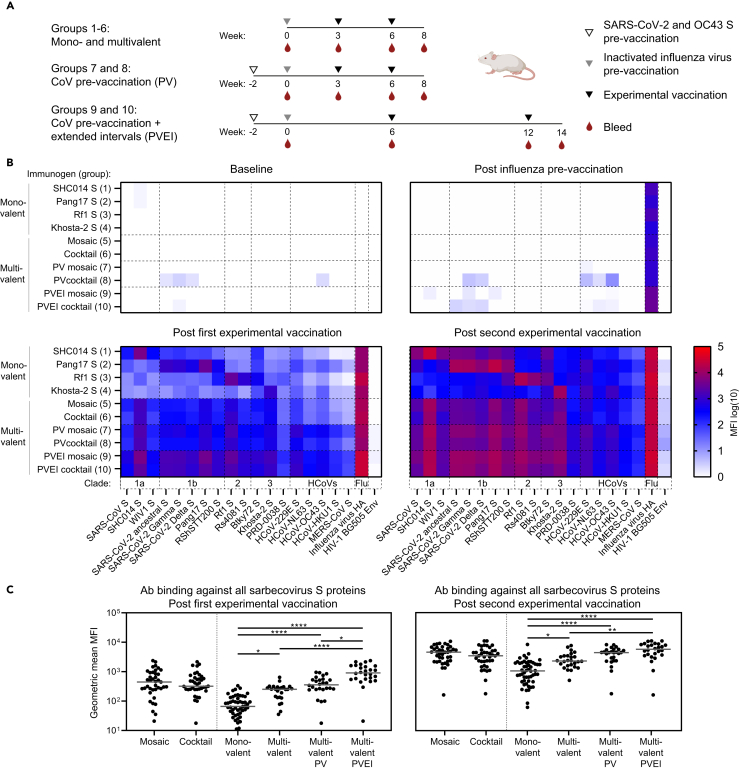
Mosaic and cocktail S-virosome vaccinations induce broad sarbecovirus Ab responses (A) Study schedules for the mono- and multivalent vaccination protocol (groups 1–6), the multivalent CoV-pre-vaccination protocol (PV: groups 7 and 8), and the multivalent CoV-pre-vaccinated extended interval protocol (PVEI: groups 9 and 10). The open triangles indicate the time of SARS-CoV-2 S and hCoV-OC43 S pre-vaccination, while the gray triangles indicate the moment of inactivated influenza virus pre-vaccination. The black triangles mark the time of the experimental vaccination and the red drops represent the bleeds used for immunogenicity assessment. (B) Ab binding responses in sera assessed using a custom Luminex assay using nine animal sarbecovirus S proteins, the S proteins of the seven human coronaviruses, the SARS-CoV-2 Gamma and Delta variant S proteins, influenza virus HA, and HIV-1 Env as a negative control. The serum samples were diluted 1:10,000. Heatmaps of Ab binding responses (in median fluorescence intensity [MFI]) at baseline (week 0), post influenza pre-vaccination (week 3/6), post the first experimental vaccination (week 6/12), and post the second experimental vaccination are shown (week 8/14). The plotted values represent the medians of the MFI values of the individual animals in each group for each protein. The background signal obtained with naked beads was subtracted. (C) Geometric mean (GM) of MFI values for Ab binding to all sarbecovirus S proteins. Each dot represents the GM MFI of one animal against all proteins. The bars indicate medians over these values across all animals. The GM MFIs between groups were compared using the Kruskal-Wallis test, followed by Dunn’s post-test (∗, *p* < 0.05; ∗∗, *p* < 0.01; ∗∗∗∗, *p* < 0.001). See also Figures S3–S6, and S8.

BALB/c mice were divided in ten groups of twelve mice each. On day 0, all mice received a pre-vaccination with an inactivated influenza vaccine to mimic an influenza-experienced immune system. Groups 1–4 received monovalent virosome formulations with either SHC014, Pang17, Rf1, or Khosta-2 S-virosome at weeks 3 and 6, and were bled at the days of vaccination and two weeks after the final vaccination, at week 8 (Figure 2A). Groups 5 and 6 received the mosaic S-virosome containing all four S proteins and the S-virosome cocktail of the four monovalent virosomes, respectively. The immunization protocol for groups 5 and 6 was the same as for groups 1–4 (Figure 2A).

In humans, a pan-sarbecovirus vaccine would not be employed in a CoV-inexperienced setting as most humans have been through SARS-CoV-2 infection and/or have received one or more vaccinations. Furthermore, most humans have gone through one or multiple episodes of infection with a common cold human CoV (hCoV). Studies have shown cross-reactivity of Abs between the different CoVs, and an increase in hCoV Ab titers after SARS-CoV-2 infection or vaccination.^35^ Some memory B cells generated through SARS-CoV-2 infection or vaccination, or through hCoV infections might be recruited by a pan-sarbecovirus vaccine, and mature further to become broadly active. In an attempt to mimic pre-existing CoV immunity, groups 7 and 8 received a pre-vaccination at week −2 with 1 μg soluble trimeric SARS-CoV-2 S and hCoV-OC43 S formulated with polyinosinic-polycytidylic acid (poly-IC) as the adjuvant.^36^ Otherwise, groups 7 and 8 were identical to groups 5 and 6, thus receiving mosaic S-virosome or S-virosome cocktail formulations, respectively (Figure 2A). hCoV-OC43 S was selected for pre-vaccinations, because cross-reactive Abs against hCoV-OC43 were found to correlate with COVID-19 disease progression, while Ab against other hCoVs did not.^37^ Finally, groups 9 and 10 received the same immunizations as groups 7 and 8, respectively, but the virosome vaccinations were given at weeks 6 and 12, thus increasing the intervals between the pre-vaccinations and the first experimental vaccinations (from 3 to 6 weeks for influenza pre-vaccination and from 5 to 8 weeks for CoV pre-vaccination), and between the two experimental vaccinations (from 3 to 6 weeks) (Figure 2A).

To analyze binding Ab responses in sera, we set up a custom Luminex assay using all animal sarbecovirus S proteins that we generated (Figure 1B), the S proteins of the seven hCoVs, the SARS-CoV-2 Gamma and Delta variant S proteins, influenza virus HA, and HIV-1 envelope glycoprotein (Env) as a negative control (Figures S1A; S3). At week 0, none of the animals in groups 1–6 showed detectable Ab responses against any of the Luminex antigens (Figure 2B). Groups 7–10 had detectable but weak responses against the SARS-CoV-2 and OC43 S at week 0 as a result of the SARS-CoV-2 S and OC43 S pre-vaccination at week −2 (Figure S4). In contrast, strong Ab responses to influenza HA were observed in all groups at week 3 as a consequence of the influenza pre-vaccination at week 0 (Figure 2B).

After one vaccination, at week 6, the monovalent vaccines in groups 1–4 induced binding Ab responses that were strongest against the autologous S proteins, and S proteins from the clades matching the immunogens (Figure 2B). Overall, Ab responses increased after the second vaccination (Figure 2B), and differences between groups were similar compared to week 6, i.e., the preference for the matched clade antigens remained (Figure 2B). In the groups receiving mosaic S-virosome and S-virosome cocktail the Ab responses were broader than in the monovalent groups (Figure 2B). The Ab responses in groups 7 and 8 that received the SARS-CoV-2 S and OC43 S pre-vaccinations, were higher compared to the responses in groups 5 and 6, especially against the non-sarbecovirus hCoVs. The difference was observed after the first and the second immunization, but was more pronounced after the first (Figures 2B and S5A). The Ab responses in groups 9 and 10, immunized with increased intervals, were significantly higher compared to groups 7 and 8, and again more pronounced after the first immunization (Figures 2B and S5B). We observed a subtle trend indicating that mosaic S-virosomes induced improved binding Ab responses compared to the S-virosome cocktail vaccinations, especially comparing the pre-vaccinated groups 7 and 8, but the differences were not statistically significant (Figures 2B, 2C, and S8).

Overall, we conclude that mosaic and cocktail virosome vaccines induce broader sarbecovirus antibody responses compared to monovalent virosome vaccines, and that SARS-CoV-2 S plus OC43 S pre-vaccination, approximating the CoV experience of humans, as well as extension of the intervals between vaccinations augmented these responses (Figure 2C).

### Multivalent S-virosomes induce cross-neutralizing antibody responses in mice

Pseudovirus neutralization assays were performed to assess NAb responses in sera collected two weeks after the final immunizations, against autologous virus SHC014, and heterologous clade 1a sarbecoviruses SARS-CoV and WIV1, clade 1b sarbecoviruses SARS-CoV-2, Pangolin-CoV GD/19, and RaTG13, merbecoviruses Neo-CoV and MERS-CoV, and common cold coronaviruses hCoV-NL63 and hCoV-229E. NAb responses are represented as inhibitory serum dilutions at which 50% neutralization is achieved (ID_50_ values) (Table S1).

**Figure 3:**
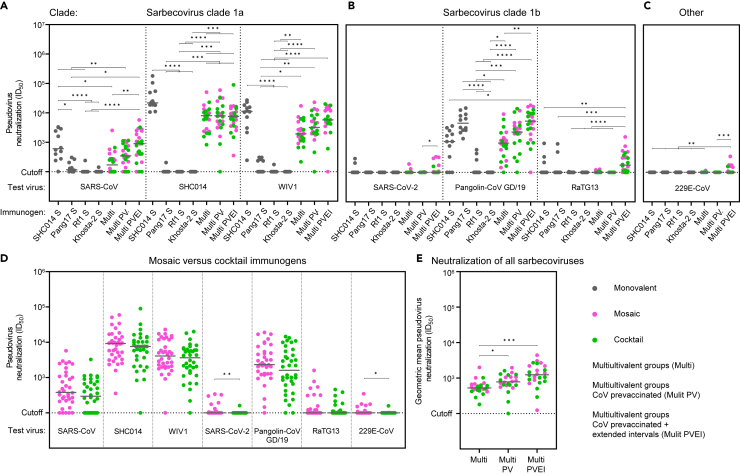
Mosaic and cocktail S-virosome vaccinations induce cross-reactive NAb responses (A) Pseudovirus neutralization of clade 1a sarbecoviruses. (B) Pseudovirus neutralization of clade 1b sarbecoviruses. (C) Pseudovirus neutralization of hCoV-229E. (D) Comparisons of mosaic S-virosome immunized animals (groups 5, 7, and 9) to S-virosome cocktail immunized animals (groups 6, 8, and 10) in their ability to neutralize diverse CoVs. The titers between groups were compared using the Mann-Whitney U test (∗, *p* < 0.05; ∗∗, *p* < 0.01). (E) Sarbecovirus neutralization breadth. For each animal in groups 5–10, the GM of neutralizing responses against all the sarbecoviruses are plotted. (A–D) Each dot represents an ID_50_ titer of an individual serum sample against a virus. (A–E) The horizontal bars indicate the medians. (A–C and E) The titers between groups were compared using the Kruskal-Wallis test, followed by Dunn’s post-test (∗, *p* < 0.05; ∗∗, *p* < 0.01; ∗∗∗, *p* < 0.001; ∗∗∗∗, *p* < 0.001). See also Figures S7 and S8, and Table S1.

SHC014 S-virosome vaccination induced potent autologous NAb responses (median ID_50_ value of 21,880), and induced cross-neutralization of clade 1a viruses SARS-CoV and WIV1 (median ID_50_ values of 605 and 11,277, respectively) (Figure 3A). The SHC014 S-virosome also induced cross-neutralization of clade 1b virus Pangolin-CoV GD/19 (median ID_50_ value of 1,113) (Figure 3B). However, the clade-matched Pang17 S-virosome induced ∼4-fold higher NAb titers against Pangolin-CoV GD/19 compared to SHC014 S-virosome (median ID_50_ values of 4,651 vs. 1,113, *p* = 0.0005) (Figure 3B). Neutralizing activity against the other clade 1b viruses was barely detectable in SHC014- and Pang17 S-virosome immunized animals (2/12 and 0/12 mice, ID_50_ values between 209 and 294, against SARS-CoV-2; 3/12 and 2/12 mice, ID_50_ values between 218 and 969, against RaTG13, respectively) (Figure 3B). Pang17 S-virosome induced significantly lower NAb titers compared to SHC014 S-virosome against clade 1a viruses (median ID_50_ values of 117 vs. 605, *p* = 0.0002 against SARS-CoV; <100 vs. 21,880, *p =* <0.0001 against SHC014; <100 vs. 11,277, *p* = <0.0001 against WIV1) (Figure 3A). A few of the animals immunized with Rf1- and Khosta-2 S-virosome (4/24 mice) cross-neutralized clade 1a and 1b sarbecoviruses (ID_50_ values between 106 and 589) (Figures 3A, 3B; Table S1). None of the monovalent S-virosome immunogens induced neutralizing responses against MERS-CoV, Neo-CoV, hCoV-229E, and hCoV-NL63 (ID_50_ values <100) (Figures 3C, S7A, and S7B). Overall, potent heterologous responses elicited by SHC014- and Pang17 S-virosomes were restricted to viruses from the clade matching the immunogen, although we note that we were unable to assess neutralization of clade 2 and 3 viruses due to the absence of a functional neutralization assay. SHC014 S-virosome elicited stronger cross-clade neutralization compared to Pang17 S-virosome.

The multivalent mosaic S-virosome and S-virosome cocktail immunogens induced somewhat weaker NAb responses compared to the monovalent S-virosomes against the clade matched viruses. This difference was most apparent comparing the responses in combined groups 5 and 6 to the responses in the monovalent groups (clade 1a: median ID_50_ values of 171 vs. 605, *p* = 0.0004 against SARS-CoV; 8,023 vs. 21,880, *p* = <0.0001 against SHC014; 1,935 vs. 11,277, *p* = 0.0007 against WIV1; clade 1b: <100 vs. <100, *p =* 0.5429 against SARS-CoV-2; 984 vs. 4,651, *p* = <0,0001 against Pangolin-CoV GD/19; <100 vs. <100, *p =* 0.6811 against RaTG13) (Figures 3A and 3B). The overall decrease in NAb titers in groups 5 and 6 was likely caused by the reduced dose of each S by 75% compared to the dose in the respective monovalent vaccines. A few animals immunized with the mosaic S-virsosome and S-virosome cocktail neutralized viruses beyond the sarbecovirus group, i.e., MERS-CoV and hCoV-229E (4/24 mice, ID_50_ values between 110 and 142) (Figures 3C and S7A). None of the animals neutralized Neo-CoV and hCoV-NL63 (ID_50_ values <100) (Figures S7A and S7B).

Immunizing with the mosaic S-virosome (groups 5, 7, and 9) induced a trend of improved NAb titers compared to the S-virosome cocktail (groups 6, 8, and 10). These differences were statistically significant against some viruses, in particular SARS-CoV-2 and hCoV-229E (Figures 3D and S7C). Details on the individual comparisons between mosaic S-virosome-vaccinated groups and S-virosome cocktail-vaccinated groups are provided in Figures S7D–S7F. Given the small differences, mosaic S-virosome and S-virosome cocktail immunized animals, which were immunized with the same vaccination strategy, were combined in the following analyses (groups 5 and 6; groups 7 and 8; groups 9 and 10).

The SARS-CoV-2 S and hCoV-OC43 S pre-vaccinated animals immunized with the multivalent vaccines (groups 7 and 8) neutralized SHC014 with similar potency compared to animals that only received the multivalent vaccines (groups 5 and 6) (median ID_50_ values of 7,959 vs. 8,023, respectively) (Figure 3A). Pre-vaccination increased NAb titers ∼2-fold against other sarbecoviruses, but the differences were not statistically significant (median ID_50_ values of 347 vs. 171, *p =* 0.5113 against SARS-CoV; 3,211 vs. 1,935, *p =* >0.9999 against WIV1; and 2,326 vs. 984, *p =* 0.9578 against Pangolin-CoV GD/19, for groups 7 and 8 versus groups 5 and 6), with the exception of SARS-CoV-2 and RaTG13 (Figures 3A and 3B). One pre-vaccinated animal neutralized SARS-CoV-2 with an ID_50_ value of 104, and no animals neutralized RaTG13 (Figure 3B). MERS-CoV, Neo-CoV, hCoV-229E, or hCoV-NL63 were not neutralized by serum samples from both groups 7 and 8 (ID_50_ values < 100) (Figures 3C, S7A, and S7B).

The animals that were vaccinated according to the extended immunization protocol (groups 9 and 10) neutralized SHC014 with similar potency compared to groups 5 and 6, and 7 and 8 (median ID_50_ values of 7,550 vs. 8,023 and 7,959) (Figure 3A). Extending the immunization intervals increased NAb titers ∼2-fold against SARS-CoV (median ID_50_ values of 899 vs. 347, *p =* >0.9999), WIV1 (median ID_50_ values of 5,768 vs. 3,211, *p =* >0.9999), and Pangolin-CoV GD/19 (median ID_50_ values of 5,460 vs. 2,326, *p =* >0.9999) (Figures 3A and 3B). A number of sera from animals immunized according to the extended protocol neutralized SARS-CoV-2 (8/24 mice, ID_50_ values between 103 and 339; median ID_50_ <100), RaTG13 (20/24 mice, ID_50_ 111–1,587, median ID_50_ 176) and hCoV-229E (8/24 mice, ID_50_ 101–347, median ID_50_ <100) (Figures 3B and 3C). The mosaic S-virosome (group 9) induced neutralizing activity in 7/12 and 6/12 animals against SARS-CoV-2 and hCoV-229E, respectively, while the S-virosome cocktail (group 10) induced neutralizing activity in only 1/12 and 2/12 animals against these viruses (Figures S7E and S7F). MERS-CoV, Neo-CoV, and hCoV-NL63 were not neutralized by group 9 and 10 sera (Figures S7A and S7B).

The pseudovirus neutralization titers correlated well with the Ab binding MFIs for each vaccination strategy (groups 1–6, Spearman *r* = 0.5429, *p* = <0.0001; groups 7 and 8, Spearman *r* = 0.5738, *p* = <0.0001; groups 9 and 10, Spearman *r* = 0.5959, *p* = <0.0001) (Figure S8), implying that a substantial proportion of the Abs contributed to neutralization, while non-neutralizing Abs are also likely present.

Overall, we conclude that mosaic and cocktail virosome vaccines induce broader sarbecovirus neutralizing responses compared to monovalent virosome vaccines. Pre-vaccinating with SARS-CoV-2 S and hCoV-OC43 S further improved neutralizing responses, and extended vaccination intervals improved NAb titers and neutralization breadth (Figure 3E).

## Discussion

As demonstrated by the SARS-CoV epidemic and the SARS-CoV-2 pandemic, the spillover of sarbecoviruses is a serious threat to global health. Here, we designed and evaluated multivalent S-virosome-based pan-sarbecovirus vaccines. Mice were immunized with virosomes displaying stabilized S constructs based on the SHC014, Pang17, Rf1, and Khosta-2 viruses, from sarbecovirus clades 1a, 1b, 2, and 3, respectively. The S constructs were displayed individually on S-virosomes which were administered as a cocktail, and co-displayed on mosaic S-virosomes. Both vaccine formulations led to broad sarbecovirus antibody responses, and these were enhanced by pre-vaccination with soluble SARS-CoV-2 S and OC43 S, in an effort to mimic pre-existing hCoV immunity, and by extended immunization intervals.

The rationale for exploring mosaic S-virosomes was that such mosaic immunogens might preferentially target and activate cross-reactive B cells, as cross-reactive B cells might benefit maximally from the multivalent presentation of the epitope they react with. Comparing the mosaic S-virosome and S-virosome cocktail vaccinations, a trend was apparent showing improved NAb titers and increased neutralizing breadth induced by the mosaic S-virosome vaccine compared to the cocktail, although this was not statistically significant. In other studies, mosaic and cocktail NP immunogens displaying SARS-CoV-2 variant S proteins induced similar responses,^38^^,^^39^^,^^40^ while mosaic NP immunogens against hepatitis C virus (HCV) and influenza virus, performed marginally better than cocktail NP formulations.^41^^,^^42^ Antigen diversity could play a role in the effectiveness of mosaic NPs. SARS-CoV-2 variant S antigens, which are relatively similar, did not benefit from mosaic NP display, while diverse sarbecovirus S antigens, diverse HCV envelope proteins, and influenza HA antigens did. However, the currently available evidence is not strongly in favor of one formulation over the other.

Other studies evaluating pan-sarbecovirus vaccines were all conducted in naive immune backgrounds.^43^ As most humans have been through SARS-CoV-2 and common cold hCoV infections and/or have received one or more vaccines, a pan-sarbecovirus vaccine would likely be employed in a CoV-experienced immune setting. Mimicking a CoV-experienced immune system, by pre-vaccinating mice with soluble SARS-CoV-2 S and hCoV-OC43 S, improved Ab binding responses after the experimental vaccinations, especially against hCoVs. The pre-vaccinations also improved NAb titers against heterologous sarbecoviruses. The improved responses can likely be attributed to antigen-specific pre-existing B cells. These data show that boosting an existing coronavirus immune response, can improve Ab responses against heterologous viruses. Other studies investigating heterologous booster vaccinations with SARS-CoV-2 variants have observed responses indicative of immune imprinting or original antigenic sin (OAS).^44^ Here, the use of divergent strains might overcome OAS. However, we note that the pre-existing SARS-CoV-2 and hCoV-OC43 responses, achieved with 1 μg S protein of each in our experiment, were rather weak as we tried to balance between generating pre-existing B cells while avoiding high titer circulating Ab responses that could interfere with the experimental vaccinations. It is likely that pre-existing B cell responses in the human population are considerably stronger than in our mouse experiment.

Heterologous NAb responses and NAb breadth were improved by extending the intervals between vaccinations. Experimental vaccinations were performed six weeks apart instead of three weeks. NAb titers improved against all heterologous sarbecoviruses tested, including SARS-CoV-2 and RaTG13, which were not or barely neutralized by animals in the other study groups (i.e., with short intervals). Similarly, neutralization of alphacoronavirus hCoV-229E was improved by the extended vaccination intervals. Extended intervals between SARS-CoV-2 vaccinations, and vaccination and SARS-CoV-2 infection, have been shown to enhance immunity in humans.^45^^,^^46^ It has been demonstrated that germinal center reactions can last for months, during which Ab somatic hypermutation of B cells continue to accumulate.^47^ Extended intervals between vaccinations can allow for improved antibody diversification and evolution. These data highlight the importance of correctly timing and spacing booster vaccinations.

In conclusion, we have shown that a multivalent sarbecovirus S-virosome vaccine can induce broad sarbecovirus Ab responses in mice, and we have identified several factors that can augment such responses. Furthermore, we have identified four SARSr-CoV S proteins, one of each sarbecovirus clade, that are useful antigens to be exploited in other vaccine platforms. Our data point out that a pan-sarbecovirus vaccine, which would be a useful tool for pandemic preparedness, is a very achievable goal.

### Limitations of the study

There are certain limitations to our study. Firstly, our focus was on evaluating the neutralizing capacity of the Abs induced by the experimental vaccinations. We choose to do so because of the established strong correlation between neutralization titers and protection, in both animals and humans.^9^^,^^48^^,^^49^^,^^50^^,^^51^ However, with no exploration into the polyfunctional role of these Abs. While it is known that COVID-19 SARS-CoV-2-positive sera can also mediate complement deposition and the killing of infected cells through antibody-dependent cellular cytotoxicity (ADCC),^52^ the polyreactivity of humoral immunity targeting sarbecoviruses was not specifically addressed in our investigation. Notably, it has been suggested that previous exposure to hCoVs may influence the polyreactivity of humoral immunity targeting SARS-CoV-2, which could be of particular interest in this kind of study.^53^

Secondly, it has been established that T cells can cross-recognize variant SARS-CoV-2 S proteins, even when recognition by memory B cells and NAbs is significantly diminished.^54^^,^^55^ This implies that T cells cross-recognizing sarbecovirus S proteins or even hCoV S proteins could play a role in the efficacy of pan-sarbecovirus and pan-CoV vaccines. However, our study did not assess T cell responses.

Lastly, we did not perform viral challenge studies. Based on the established strong correlations between NAbs and protection (see aforementioned text),^9^^,^^48^^,^^49^^,^^50^^,^^51^ we inferred that high levels of NAbs would be protective. Indeed previous research has demonstrated that sarbecovirus S vaccines can protect mice against viral challenge, when NAb titers are similar to those observed in our study.^14^^,^^56^

## STAR★Methods

### Key resources table

**Table:** 

REAGENT or RESOURCE	SOURCE	IDENTIFIER
**Antibodies**		

COVA2-02	(Brouwer et al., 2020)^30^	N/A
COVA1-16	(Brouwer et al., 2020)^30^	N/A
S309	(Pinto et al., 2020)^32^	N/A
J08	(Andreano et al., 2021)^31^	N/A
Goat anti-mouse IgG-PE	Southern Biotech	Cat# 1030-09, 1030-09S; RRID:AB_2794297, RRID:AB_2794298

**Chemicals, peptides, and recombinant proteins**		

PBS	Thermo Fisher	Cat# 10010023
PEI MAX	Polysciences	Cat# 24765-1
3,3′,5,5′-tetranethylbenzidine	Sigma-Aldrich	Cat# T4444
Poly-L-Lysine Hydrobromide	Sigma-Aldrich	Cat# P1399
Penicillin	Sigma-Aldrich	Cat# P3032-10MI
Streptomycin	VWR	Cat# 382-EU-100G
Gibco™ MEM Non-Essential Amino Acids Solution (100X)	Thermo Fisher	Cat #11140050
Gibco™ HEPES 1M	Thermo Fisher	Cat #15630056
Blasticidin solution	Invivogen	Cat #ant-bl-05
SARS-CoV-2 S Foldon his	This Study	N/A
SARS-CoV-2 Gamma S Foldon his	This Study	N/A
SARS-CoV-2 Delta S Foldon his	This Study	N/A
SARS-CoV-2 Gamma S Foldon his	This Study	N/A
SARS-CoV S Foldon his	This Study	N/A
SHC014 S Foldon his	This Study	N/A
WIV1 S Foldon his	This Study	N/A
Pang17 S Foldon his	This Study	N/A
RShST200 S Foldon his	This Study	N/A
Rf1 S Foldon his	This Study	N/A
Rs4081 S Foldon his	This Study	N/A
Btky72 S Foldon his	ARCO Biosystems	SPN-S52Hu
Khosta-2 S Foldon his	This Study	N/A
PRD-0038 S Foldon his	This Study	N/A
HCoV-229E S Foldon his	This Study	N/A
HCoV-NL63 S Foldon his	This Study	N/A
HCoV-OC43 S Foldon his	This Study	N/A
HCoV-HKU1 S Foldon his	This Study	N/A
MERS-CoV S Foldon his	This Study	N/A
Influenza virus HA	The Native Antigen Company	REC32023-100
HIV-1 BG505 Env	(Sanders et al., 2023)^57^	N/A
Influenza virus virosomes	Mymetics B.V.	N/A
3M-052	3M	N/A
QS-221	MedChem Express	Cat# HY-101092
Polyinosinic-polycytidylic acid	Invivogen	vac-pic

**Critical commercial assays**		

Nano-Glo Luciferase Assay System	Promega	Cat# N1130
4-16% Bis-Tris NuPAGE gels	Invitrogen	BN1002BOX
Nucleobond Xtra Maxi kit	Macherey-Nagel	Cat# 740414.50

**Experimental models: Cell lines**		

FreeStyle 293F cells	Thermo Fisher	Cat# R79007
HEK 293T/ACE2 cells	(Schmidt et al., 2022)^61^	N/A
HEK 293T cells	ATCC	Cat# CRL-11268
Huh7 cells	A gift from dr. François-Loïc Cosset	N/A

**Experimental models: Organisms/strains**		

BALB/cAnNCrl mice	Charles River Laboratories	N/A

**Recombinant DNA**		

pHIV-1_NL43_ΔENV-NanoLuc plasmid	(Schmidt et al., 2022)^61^	N/A
SARS-CoV-2-S_Δ19_ plasmid	(Schmidt et al., 2022)^61^	N/A
SARS-CoV-S_Δ19_ plasmid	(Brinkkemper et al., 2022)^40^	N/A
SHC014S_Δ19_ plasmid	This study	N/A
WIV1-S_Δ19_ plasmid	This study	N/A
Pangolin-CoV-GD/19-S_Δ19_ plasmid	This study	N/A
RaTG13-S_Δ19_ plasmid	This study	N/A
229E-CoV-S_Δ19_ plasmid	This study	N/A
MERS-CoV-S_Δ19_ plasmid	This study	N/A
Neo-CoV-S_Δ19_ plasmid	This study	N/A
hCoV-NL63 plasmid	Addgene	#172666
SARS-CoV-2-S-Foldon-his plasmid	(Brouwer et al., 2020)^30^	N/A
SARS-CoV-2-Gamma-S-Foldon-his plasmid	This study	N/A
SARS-CoV-2-Delta-S-Foldon-his plasmid	This study	N/A
SARS-CoV-S-Foldon-his plasmid	This study	N/A
SHC014-S-Foldon-his plasmid	This study	N/A
WIV1-S-Foldon-his plasmid	This study	N/A
Pang17-S-Foldon-his plasmid	This study	N/A
RshSTT200-S-Foldon-his plasmid	This study	N/A
Rf1-S-Foldon-his plasmid	This study	N/A
Rs4081-S-Foldon-his plasmid	This study	N/A
Khosta-2-S-Foldon-his plasmid	This study	N/A
PRD-0038-S-Foldon-his plasmid	This study	N/A
229E-S-Foldon-his plasmid	(Grobben et al., 2022)^35^	N/A
NL63-S-Foldon-his plasmid	(Grobben et al., 2022)^35^	N/A
OC43-S-Foldon-his plasmid	(Grobben et al., 2022)^35^	N/A
HKU1-S-Foldon-his plasmid	(Grobben et al., 2022)^35^	N/A
MERS-CoV-S-Foldon-his plasmid	(Grobben et al., 2022)^35^	N/A
BG505-Env plasmid	(Sanders et al., 2023)^57^	N/A

**Software and algorithms**		

GraphPad Prism v8	GraphPad	N/A
UCSF ChimeraX	(Goddard et al., 2018)^64^	N/A
Adobe Illustrator	Adobe	N/A

**Other**		

Superose 6 increase 10/300 GL	Sigma-Aldrich	Cat# GE29-0915-96
Octet K2 system	Sartorius (FortéBio)	N/A
Octet Biosensors: Protein A	Sartorius (FortéBio)	Cat# 18-5010
Vivaspin 20, 100.000 kDa MWCO, Polyethersulfone	Sigma-Aldrich	Cat# GE28-9323-63
Fast Digest BamHI	Thermo Scientific	Cat# FD0054
Fast Digest Green buffer 10x	Thermo Scientific	Cat# B72
Fast Digest PstI	Thermo Scientific	Cat# FD0614
FreeStyle 293 Expression medium	Thermo Scientific	Cat# 12338018
DMEM	Sigma-Aldrich	Cat# D6429-500ML
Glutamax supplement	Thermo Fisher	Cat# 35050061
Steritop Filter Units	Merckmillipore	Cat# C3239
Glomax	Turner BioSystems	Model# 9101-002
Microplate 96 well half area white	Greiner bio-one	Cat# 675074
AKTA Avant150 FPLC system	Cytiva	N/A
MagPlex-C Microspheres	DiaSorin	MC100xx
MAGPIX system	DiaSorin	MAGPIX-XPON4.1-CEIVD

### Resource availability

#### Lead contact

Further information and requests for resources and reagents should be directed to and will be fulfilled by the lead contact, Rogier W. Sanders (r.w.sanders@amsterdamumc.nl).

#### Materials availability

All reagents will be made available on request after completion of a Materials Transfer Agreement.

#### Data and code availability

• The data supporting the findings of the study are available from the corresponding authors upon reasonable request.
• This paper does not report original code.
• Any additional information required to reanalyze the data reported in the paper is available from the lead contact upon request.

### Experimental models and subject details

#### Cell lines

The HEK293T (ATCC CRL-11268) and HEK293F (Life Technologies) cell lines are human female embryonic kidney cells engineered to enhance the production of recombinant protein or retrovirus. HEK293F cells are designed for suspension growth. HEK293T cells were maintained in flasks with DMEM + 10% FBS + 1% penicillin-streptomycin at 37°C with 5% CO_2_. HEK293F cells were cultivated in 293FreeStyle expression medium (Life Technologies) at 37°C with 8% CO_2_ and agitation at 125 rpm. HEK293T/ACE2 represents a human embryonic kidney cell line expressing Human Angiotensin-Converting Enzyme 2. HEK293T/ACE2 cells were cultured in flasks with DMEM + 10% FBS + 1% penicillin-streptomycin + 5 μg/ml blasticidin at 37°C with 5% CO_2_. Huh7 cells were maintained in DMEM + 10% FBS + 1% penicillin-streptomycin + 1x MEM NEAA + 1mM HEPES at 37°C with 5% CO_2_.

#### Mice

Eight-week-old BALB/cAnNCrl mice, sourced from Charles River Laboratories, were procured and accommodated at the Animal Research Institute Amsterdam, maintaining BSL-2 conditions. Adhering to the Dutch Experiment on Animals Act, all experiments received approval from the Animal Ethics Committee of the Amsterdam UMC under Permit number 202011565.

### Method details

#### Construct design

The SARS-CoV-2-S-foldon-his plasmid was described before.^30^ In brief, the gene encoding SARS-CoV-2 S residues 1-1138 (Wuhan-Hu-1; GenBank MN908947.3) was cloned into a pPPI4 backbone containing a foldon T4 trimerization domain followed by a hexahistidine-tag with PstI-BamHI digestion and ligation. This construct contains proline substitutions at 986 and 987, and a GGGG substitution at the furin cleavage site (682–685). To generate the SARS-CoV-2 variant prefusion S the following mutations were included: Gamma: L18F, T20N, P26S, D138Y, R190S, K417T, E484K, N501Y, D614G, H655Y, and T1027I; Delta: T19R, G142D, E156G, Δ157-158, L452R, T478K, D614G, P681R and D950N. To make the SARS-CoV (GenBank AAP33697.1), SHC014 (GenBank KC881005), WIV1 (GenBank KF367457), Pang17 (GenBank QIA48632), RshSTT200 (GISAID EPI_ISL_852605), Rf1 (GenBank DQ412042), Rs4081 (GenBank KY417143), Khosta-2 (GenBank MZ190138) and PRD-0038 (GenBank MT726045) prefusion S constructs, the S genes were aligned to the SARS-CoV-2 S gene and the 986 and 987 proline substitutions and the 1138 truncation were directly copied. DNA constructs were synthesized at Thermo Fisher Scientific and cloned into the pPPI4 foldon-his backbone using Gibson assembly. The hCoV-229E, NL63, hCoV-OC43, hCoV-HKU1 and MERS-CoV constructs were described previously,^35^ as was the HIV-1 BG505 Env construct.^57^

#### Protein expression and purification

HEK293F cells (0.8-1.2 million cells per mL) were transiently transfected with the coronavirus S constructs. Cells were maintained in Freestyle medium (Life Technologies). A mix of PEImax (937.5 μg/L cells) and expression plasmid (312.5 μg/L cells) was prepared in OptiMEM (Gibco) and added to the cells. Six days after the transfection the supernatants were collected by centrifuging the cell cultures at 3,000 x g for 30 min and were filtered through 0.22 μm Steritop filters (Merck Millipore). Supernatants were subjected to Ni-NTA agarose beads for affinity purification. Proteins were eluted and buffer exchanged to PBS and concentrated using Vivaspin filters (GE Healthcare) with a 100,000 Da cut-off. Additionally, proteins were applied to a Superose 6 increase 10/300 GL column (GE healthcare) in PBS for size exclusion chromatography. Appropriate size fractions were collected and subsequently pooled and concentrated using Vivaspin filters if necessary. Concentrations were measured using the peptidic molecular weight with Nanodrop. Proteins were stored at -80°C. The BtKY72 S protein was acquired from ACRO Biosystems, while influenza A [A/Victoria/2570/2019 (H1N1)pdm09-like virus] hemagglutinin was obtained from The Native Antigen Company. HIV-1 BG505 Env was produced as described elsewhere.^57^

#### Blue Native Page analysis

4-16% Bis-Tris NuPAGE gels (Invitrogen) were loaded with 3 μg of S protein mixed with loading dye and run at 200V for approximately 1.5 h.

#### Bio-layer inferometry (BLI)

S proteins were diluted to 100 nM in PBS with 0.1% bovine sera albumin and 0.02% Tween 20. Antibody binding was assessed using a ForteBio Octet K2. Assays were performed at 30°C with agitation set at 1,000 rpm. Antibodies were loaded on protein A sensors (ForteBio) at 10 mg/mL in PBS with 0.1% bovine sera albumin and 0.02% Tween 20 until a binding threshold of 1 nm was reached. Association and dissociation were measured for 300 s.

#### Virosome preparation

Virosomes were prepared following the previously outlined procedure.^58^ Briefly, inactivated influenza A/Victoria/2570/2019 was solubilized using the detergent octaethyleneglycol-mono(n-dodecyl)ether (OEG), and the viral nucleocapsid was removed through centrifugation. The lipids dioleoyl-phosphatidylcholine, cholesterol, and the click chemistry lipid dicyclobenzooctyl-phosphatidylethanolamine (DBCO-PE), dissolved in OEG, were then introduced to the supernatant. All lipids were sourced from Avanti Polar Lipids, USA. Subsequently, OEG was removed via batch chromatography on BioBeads SM2 (BioRad, USA) as per the described method,^59^ and the virosomes underwent sterilization through filtration. The synthesis and purification of 2-azidoethyl thiophosphodichlorate (ATPD) were carried out according to Acme Bioscience's protocol (China). S protein underwent dialysis against 50 mM HEPES pH 8.5 for 4 hours, followed by mixing with ATPD at a 200:1 ratio of ATPD to protein for 1 hour at room temperature (RT). The resulting product underwent overnight dialysis against 2,000 volumes of buffer (145 mM NaCl, 5 mM HEPES, 1 mM EDTA, pH 7.4). The resultant S-azide was filter-sterilized and then incubated with virosomes for a minimum of 24 hours at 25 °C, leading to the covalent coupling of S to virosomes through azide-DBCO-PE click chemistry. There was a three-fold excess of DBCO on the virosomal lipids available for coupling to the azide on S. The concentration of S was estimated through SDS-PAGE gels. The concentration of S was measured in each stage of the vaccine production by UPLC. Briefly, a Waters UPLC equipped with a C18 column was ran with a gradient of 50 to 95% acetonitrile/0.1% trifluoroacetic acid (TFA) in water with 0.1% TFA at 60°C for 5 minutes, with optical detection at 280 nm. A linear calibration curve could be established. Adjuvants were incorporated into the virosomal membrane through post-insertion. In this process, 3M-052 (3M, USA) was dissolved in ethanol, and a small quantity of this adjuvant was swiftly mixed with the virosomes. The mixture was then incubated for 30 minutes at RT, after which QS21 was added from a stock solution in phosphate-buffered saline (PBS), adjusted to pH 6.5. Coupling of the S antigen to the virosomes was checked for the Pangolin and SHC14 strain in an ELISA, where intact virosomes were bound to ELISA plates coated with an anti-influenza hemagglutinin antibody, and S was detected with a recombinant human ACE2-Fc construct and a secondary antibody to the Fc coupled to HRP (The other two strains do not recognize human ACE2). Throughout the ELISA, the virosomes remained intact.

#### Mouse immunizations

Balb/c mice were divided in ten groups of twelve mice each. All mice received 2 μg of inactivated influenza A/Victoria/2570/2019 vaccine at day 0. S-virosomes contained 3 μg S and two adjuvants 3M-052 and QS21, each 1 μg, per dose. Groups 1-4 received the monovalent S-virosome vaccinations at weeks 3 and 6, and were bled at the days of vaccination and at week 8. Groups 5 and 6 received the mosaic S-virosome and the S-virosome cocktail, respectively. The immunization protocol for groups 5 and 6 was the same as for groups 1-4. Groups 7 and 8 received the same as the animals in groups 5 and 6, respectively, with the addition of a 1 μg soluble SARS-CoV-2 S and hCoV-OC43 S vaccination at week -2. The soluble S proteins were adjuvanted with 50 μg polyinosinic-polycytidylic acid (poly-IC) adjuvant. Groups 9 and 10 received the same immunizations as groups 7 and 8, respectively, but the virosome vaccinations were given at week 6 and 12. Vaccinations were applied subcutaneously in the neck skin-fold. Mice were housed at the Animal Research Institute Amsterdam under BSL-2 conditions. All procedures were done in accordance with the Dutch Experiment on Animals Act and were approved by the Animal Ethics Committee of the Amsterdam UMC (Permit number 202011565) and in accordance with the ARRIVE guidelines.

#### Luminex binding assay

A custom Luminex assay was used as described previously.^35^ In short, a two-step carbodiimide reaction was used to covalently couple spike proteins to Luminex Magplex beads (Bio-rad laboratories) with a ratio of 75 μg protein to 12.5 million beads. The following proteins were coupled: SARS-CoV S, SHC014 S, WIV1 S, SARS-CoV-2 S, SARS-CoV-2 Gamma S, SARS-CoV-2 Delta S, Pang17 S, RshSTT200 S, Rf1 S, Rs4081 S, BtKY72 S, PRD-0038 S, hCoV-229E S, hCoV-NL63 S, hCoV-OC43 S, hCoV-HKU1 S, MERS-CoV S, Influenza HA and HIV-1 BG505 Env. Following the outcome of optimization experiments, sera were diluted 1:10,000, while the earliest time points (weeks -2 and 0) of groups 7-10 were diluted 1:1,000. Beads and diluted sera were incubated overnight, followed by a 2 h incubation step with goat-anti-mouse IgG-PE (Southern Biotech) for detection. The read-out was performed on a Magpix machine (Luminex). The resulting mean fluorescence intensity (MFI) values represent the median of approximately 50 beads per well and were corrected by subtraction of MFI values from wells containing buffer and beads only.

#### Pseudovirus construct design

Either one of expression plasmids, pPPI4 or pCR3, was used to generate constructs containing the spike protein of hCoV-229E, SARS-CoV, SHC014, WIV1, Pangolin-CoV GD/19, RaTG13, MERS-CoV, and Neo-CoV. These constructs were custom-synthesized as Gblocks which were incorporated into expression plasmid through Gibson assembly reactions. The hCoV-NL63 construct (Addgene plasmid #172666; http://n2t.net/addgene:172666) was provided by David Nemazee,^60^ while the SARS-CoV-2 plasmid was provided by Paul D. Bieniasz.^61^ The hCoV-NL63 construct included an I507L mutation to enhance its affinity for human ACE2.^60^ Similarly, a T403R mutation was introduced to the RaTG13 S to enhance entry into ACE2 expressing cells,^62^ while the Neo-CoV construct contained a T507F mutation designed to facilitate entry into human ACE2 expressing cells.^63^

#### Pseudovirus production

The pseudoviruses were generated by co-transfecting the plasmid expressing the coronavirus spike protein with the pHIV-1NL43 ΔEnv-NanoLuc reporter virus plasmid in HEK293T cells, as described elsewhere.^61^ In brief, 5 μg of plasmid expressing S and 15 μg of pHIV-1NL43 ΔEnv-NanoLuc plasmid were co-incubated with 60 μg PEImax in OPTIMEM. This mixture was added to sub-confluent HEK293T cells in a T75 flask. Cell supernatant containing the pseudovirus was collected 48 h post-transfection, filtered through a 0.22 μm filter, and stored at -80°C until further use.

#### Neutralization assay

HEK293T-ACE2 cells,^61^ and Huh7 cells were seeded one day prior to the experiment at a density of 20,000 cells per well and 10,000 cells per well, respectively. Heat-inactivated sera were serial diluted in steps of 3-fold in culture medium before being mixed in a 1:1 ratio with pseudovirus. Subsequently, the mixture was incubated for 1 h at 37°C. The mixtures, containing the pseudovirus and sera, were added at a 1:1 ratio to HEK293T-ACE2 cells (for SARS CoV, SHC014, WIV1, Pangolin-CoV GD/19, SARS CoV-2, RaTG13, hCoV-NL63 and Neo-CoV) and Huh7 cells (for hCoV-229E and MERS-CoV). Following a 48-h incubation, the culture medium from each well was aspirated, cells were lysed and lysates transferred to half-area 96-wells white microplates (Greiner Bio-One). Luciferase activity was quantified using the Nano-Glo Luciferase Assay System (Promega) with a Glomax plate reader (Turner BioSystems). The average value of relative luminescence values (RLU) from duplicate wells was used to determine inhibition titers (ID_50_) by a non-linear regression using the standard [inhibitor] vs. response variable slope (four parameters) in GraphPad Prism 9.

### Quantification and statistical analysis

The midpoint neutralization titers were determined using Graphpad Prism 8.0. Comparisons between two experimental groups were made using a Mann-Whitney U test, and comparisons between multiple experimental groups were made using the Kruskal-Wallis test and Dunn’s post-test (∗, *p* < 0.05; ∗∗, *p* < 0.01; ∗∗∗, *p* < 0.001; ∗∗∗∗, *p* < 0.0001).
