# Supplementary material for: A spike virosome vaccine induces pan-sarbecovirus antibody responses in mice
Source: iScience. 2024 Apr 10;27(5):109719. doi: 10.1016/j.isci.2024.109719 (PMC11068555; doi:10.1016/j.isci.2024.109719)
Supplement: Document S1. Figures S1–S8 [file mmc1.pdf]

iScience, Volume *27*

Supplemental information

A spike virosome vaccine induces pan-sarbecovirus antibody responses in mice

Mitch Brinkkemper, Meliawati Poniman, Esther Siteur-van Rijnstra, Widad Ait Iddouch, Tom P.L. Bijl, Denise Guerra, Khadija Tejjani, Marloes Grobben, Farien Bhoelan, Denzel Bemelman, Ronald Kempers, Marit J. van Gils, Kwinten Sliepen, Toon Stegmann, Yme U. van der Velden, and Rogier W. Sanders

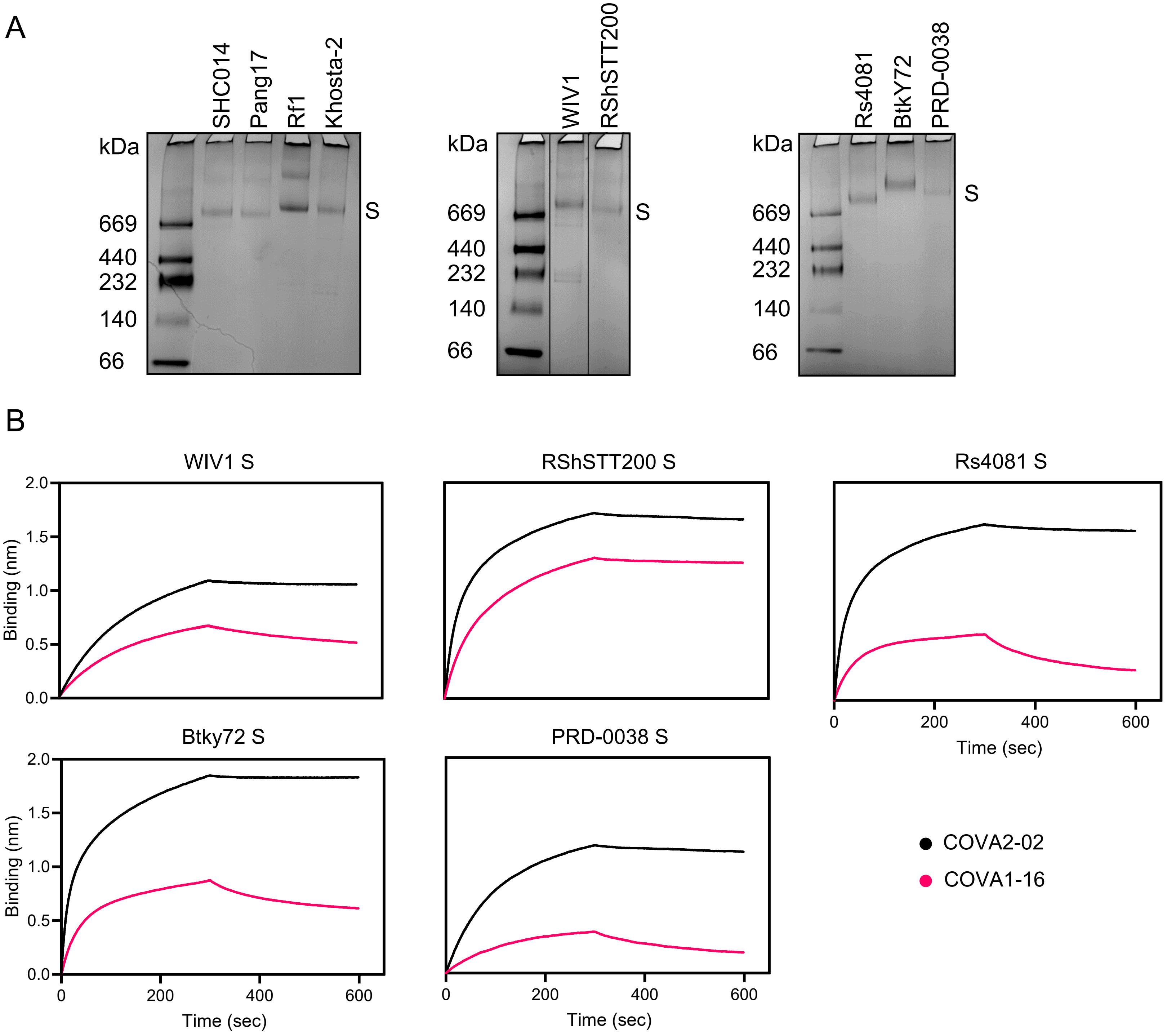

Figure S1. Sarbecovirus S characterization, related to figure 1 (A) Blue-Native PAGE gels of purified S proteins. (B) BLI sensorgrams of binding reactions of monoclonal Abs isolated from SARS-CoV-2 survivors to sarbecovirus S

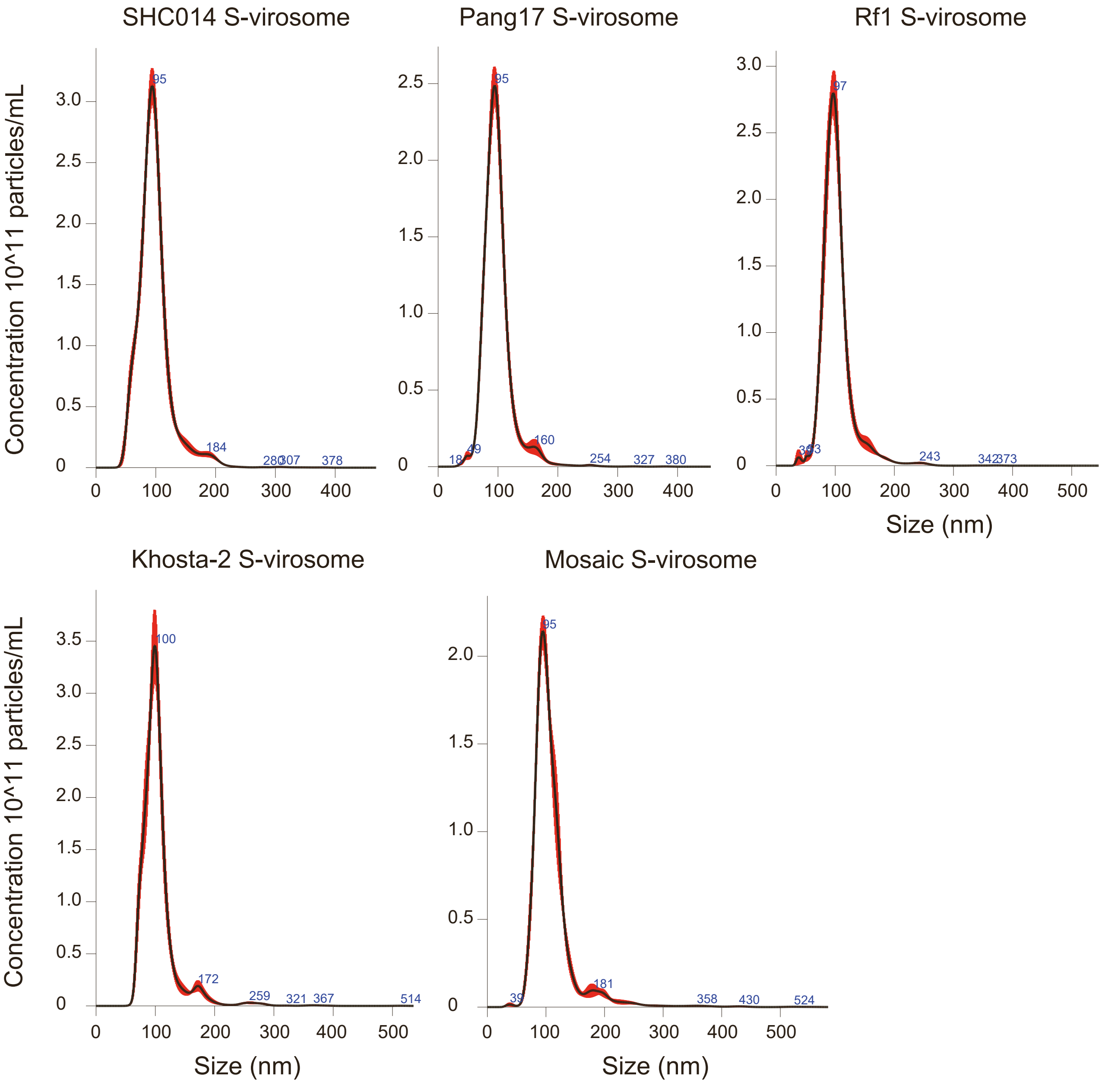

Figure S2. S-virosome size distribution, related to figure 1 Virosome size distribution as measured by nanosight.

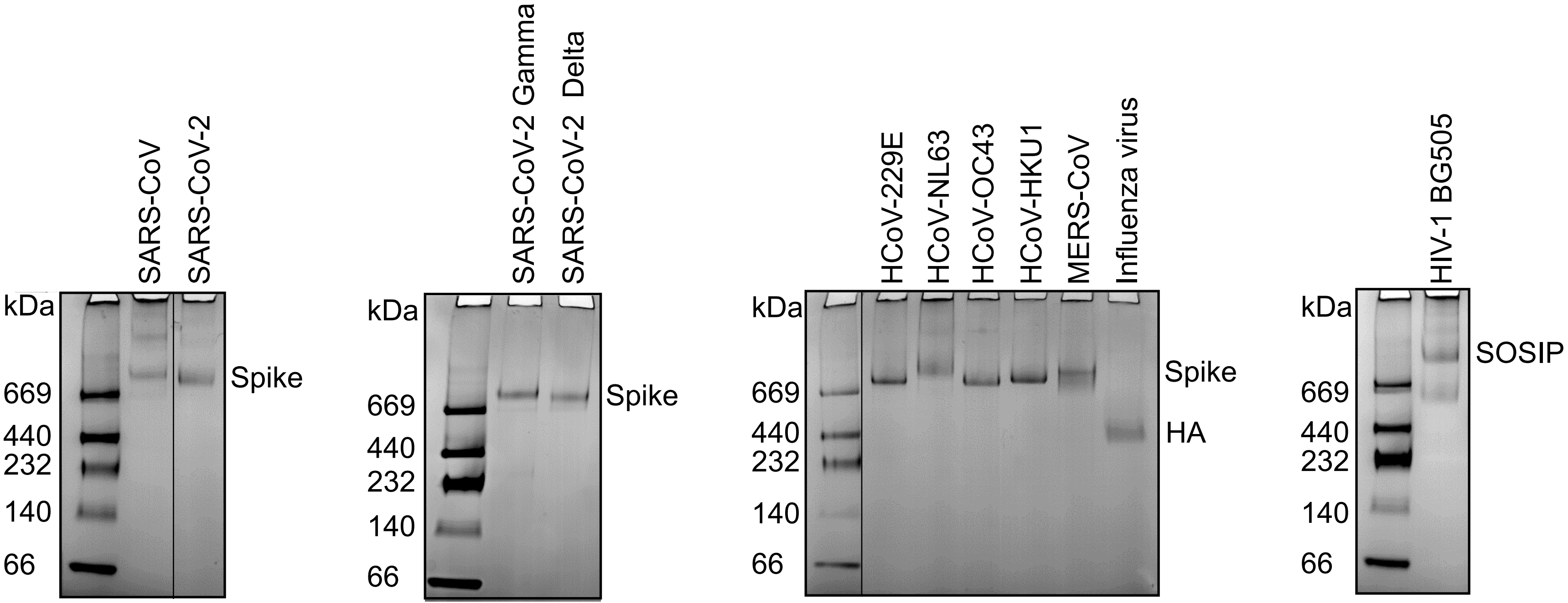

Figure S3. Luminex assay protein panel characterization, related to figure 2 Blue-Native PAGE gels of purified glycoproteins used in the custom Luminex assay.

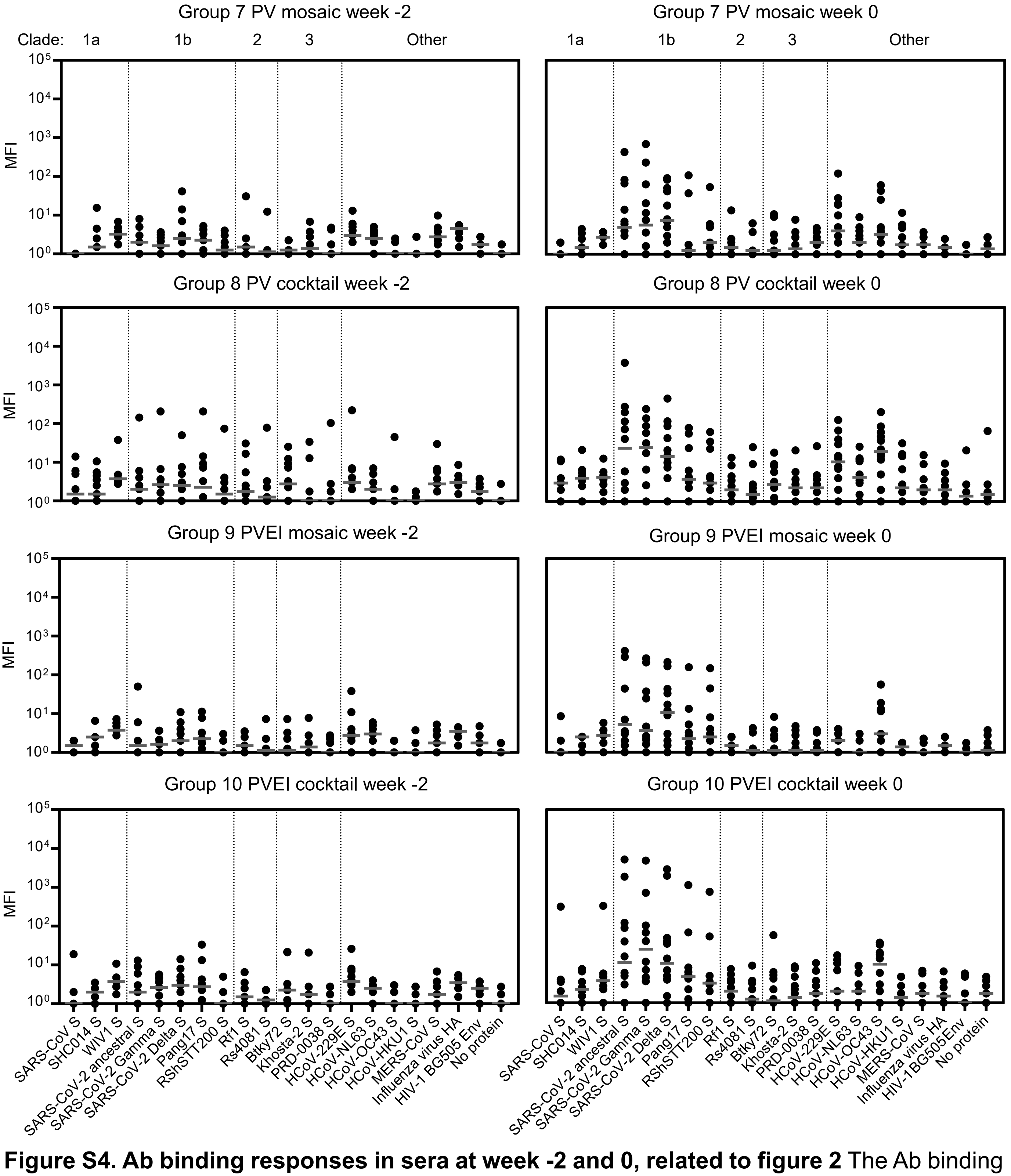

Figure S4. Ab binding responses in sera at week -2 and 0, related to figure 2 The Ab binding responses in sera from groups 7-10 at weeks -2 and 0, measured in a custom Luminex assay. The serum samples were diluted 1:1,000. The dots represent the MFI values of individual serum samples tested against each protein. The horizontal bars indicate the medians.

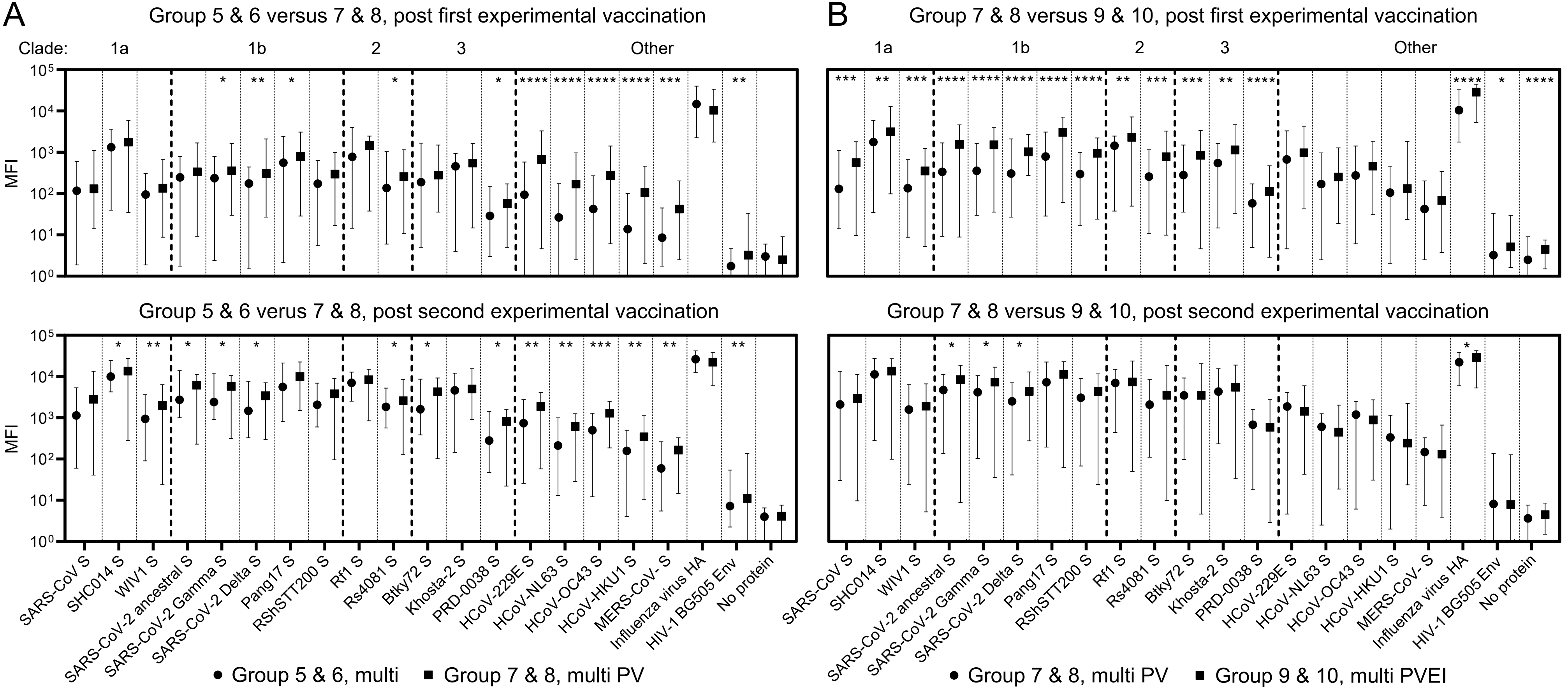

Figure S5. Ab binding responses in sera, comparing different vaccination protocols, related to figure 2 (A) Comparison of mice that received the multivalent vaccines to mice that received the multivalent vaccines and were pre-vaccinated. The Ab binding responses post the first experimental vaccination (week 6) (upper), and the Ab binding responses post the second experimental vaccination (week 8) (lower) (B) Comparison of mice that received the multivalent vaccines and were pre-vaccinated to mice that received the multivalent vaccines and were pre-vaccinated, with extended vaccination intervals. The Ab binding responses post the first experimental vaccination (week 6) (upper), and the Ab binding responses in mice post the second experimental vaccination (lower). (A,B) The Ab binding responses were measured in a custom Luminex assay. The sera were diluted 1:10,000. The dots represent the median MFI values. The whiskers indicate the range of MFIs measured in all serum samples. The MFI values between groups were compared using the Mann-Whitney U test (*, $p < 0.05$; **, $p < 0.01$; ***, $p < 0.001$; ****, $p < 0.001$).

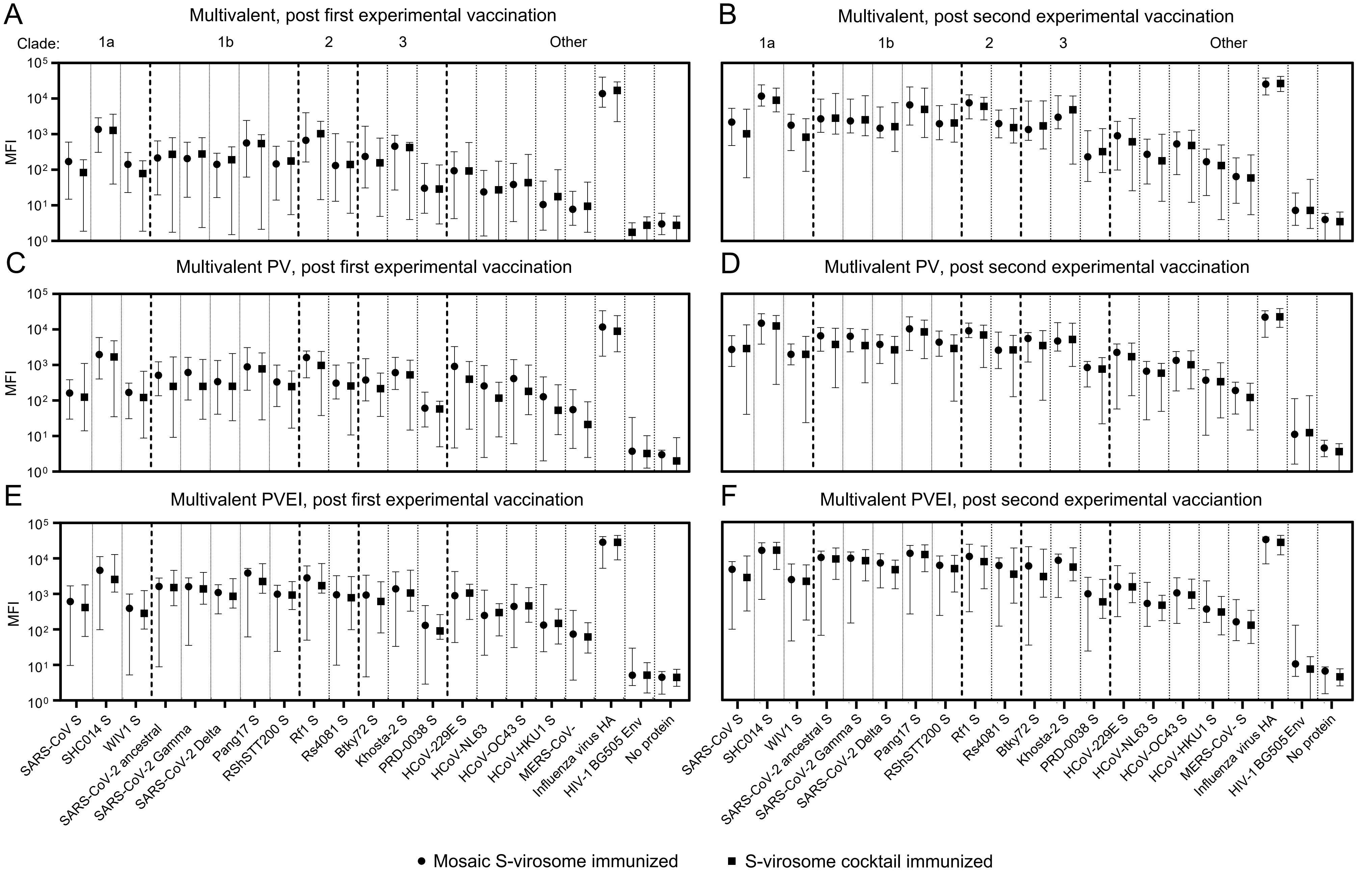

Figure S6. Ab binding responses in sera, comparing mosaic S-virosome and S-virosme cocktail vaccinations, related to figure 2 (A) Comparison of Ab binding responses in mosaic S-virosome versus cocktail S-virosome vaccinated mice post the first experimental vaccination (week 6). (B) Comparison of Ab binding responses in mosaic S-virosome versus cocktail S-virosome vaccinated mice post the second experimental vaccination (week 8). (C) Comparison of mosaic S-virosome versus cocktail S-virosome vaccinated mice, mice received the CoV-pre-vaccination post the first experimental vaccination (week 6). (D) Comparison of mosaic S-virosome versus cocktail S-virosome vaccinated mice, mice received the CoV-pre-vaccination post the second experimental vaccination (week 8). (E) Comparison of mosaic S-virosome versus cocktail S-virosome vaccinated mice, mice received the CoV-pre-vaccination, and were vaccinated with extended vaccination intervals post the first experimental vaccination (week 6). (F) Comparison of mosaic S-virosome versus cocktail S-virosome vaccinated mice, mice received the CoV-pre-vaccination, and were vaccinated with extended vaccination intervals post the second experimental vaccination (week 8). (A-E) The Ab binding responses in sera measured in a custom Luminex assay. The sera were diluted 1:10,000. The dots represent the median MFI values. The whiskers indicate the range of MFIs measured in all serum samples. The MFIs between groups were compared using the Mann-Whitney U test.

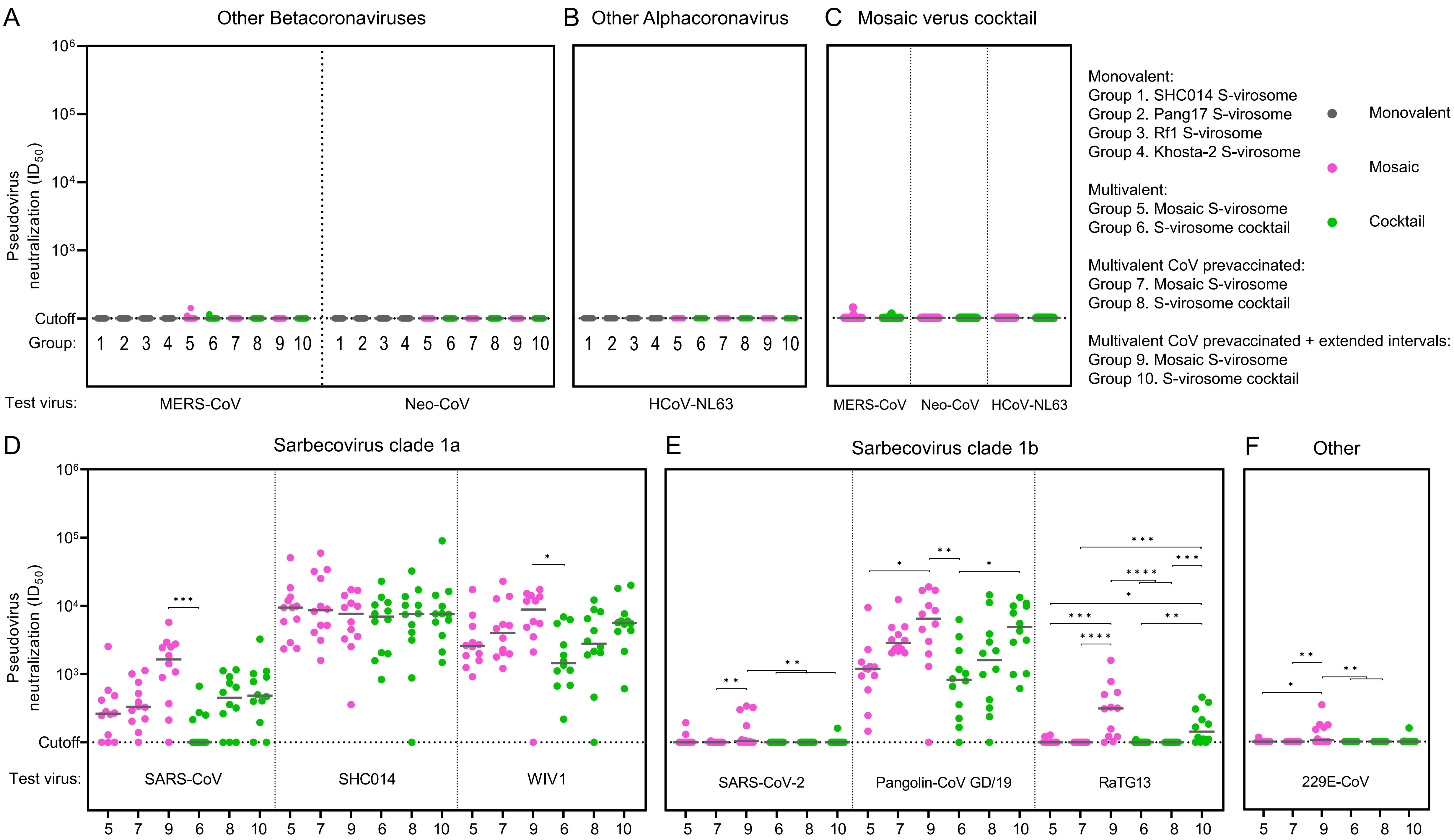

Figure S7. Pseudovirus neutralization at final bleed, related to figure 3 (A) Pseudovirus neutralization of betacoronaviruses MERS-CoV and Neo-CoV. (B) Pseudovirus neutralization of alphacoronavirus hCoV-NL63. (C) Comparison of pseudovirus neutralization by mosaic S-virosome immunized animals (groups 5, 7 and 9) to S-virosome cocktail immunized animals (groups 6, 8 and 10). (D) Pseudovirus neutralization of clade 1a sarbecoviruses by animals in groups 5-10. (E) Pseudovirus neutralization of clade 1b sarbecoviruses by animals in groups 5-10. (F) Pseudovirus neutralization of hCoV-229E by animals in groups 5-10. (A-F) The dots represent ID50 titers of individual serum samples against each virus. The horizontal bars indicate the median. The titers between groups were compared using the Kruskal-Wallis test, and Dunn's post-test (*, $p < 0.05$; **, $p < 0.01$; ***, $p < 0.001$; ****, $p < 0.001$).

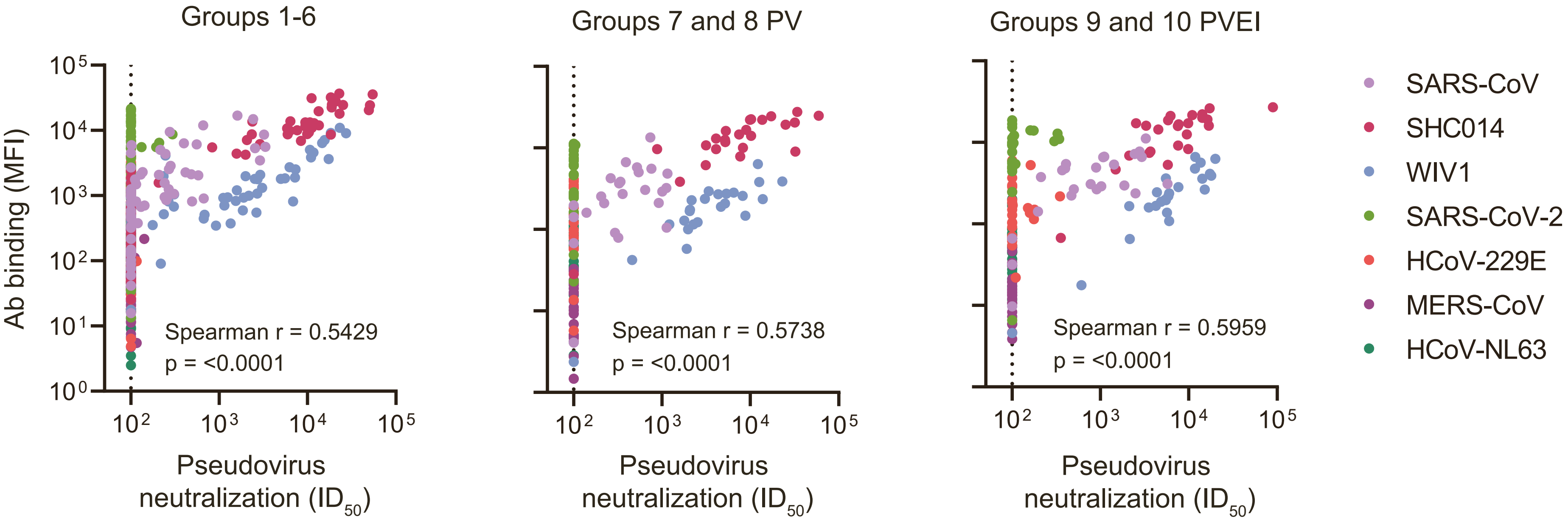

Figure S8. Correlation between pseudovirus neutralization and antibody binding, related to figures 2 and 3 Correlation between pseudovirus neutralizton and antibody binding in groups 1-6 (right), 7 and 8 (middle), and 9 and 10 (left).

Table S1. Pseudovirus neutralization ID50 titers, related to figures 3 and S7

Pseudovirus sera neutralization ID50s

			Pseudovirus Panel										
**Virosome vaccine**	**Immunization protocol**	**Mice ID**	**SARS-CoV**	**SHC014**	**WIV1**	**SARS-CoV-2 D614G**	**Pangolin-CoV GD/19**	**RaTG13 T403R**	**MERS-CoV**	**Neo CoV T510F**	**HCoV-NL63 I507L**	**HCoV-229E-CoV**
SHC014 S	Standard	gr1 - 1	241	10620	3053	<100	1111	<100	<100	<100	<100	<100
		gr1 - 2	491	25215	2156	<100	<100	<100	<100	<100	<100	<100
		gr1 - 3	655	18840	14585	<100	<100	<100	<100	<100	<100	<100
		gr1 - 4	101	18742	245	<100	<100	<100	<100	<100	<100	<100
		gr1 - 5	398	54455	10588	<100	338	<100	<100	<100	<100	<100
		gr1 - 6	2425	105332	18373	<100	3258	<100	<100	<100	<100	<100
		gr1 - 7	275	22898	27169	<100	2608	969	<100	<100	<100	<100
		gr1 - 8	1605	180763	23238	<100	1960	<100	<100	<100	<100	<100
		gr1 - 9	3218	11077	15611	294	3755	338	<100	<100	<100	<100
		gr1 - 10	3335	20861	11642	<100	1745	<100	<100	<100	<100	<100
		gr1 - 11	2879	49276	7215	209	789	218	<100	<100	<100	<100
		gr1 - 12	554	18283	10912	<100	1114	<100	<100	<100	<100	<100
Pang17 S	Standard	gr2 - 13	130	<100	265	<100	9318	929	<100	<100	<100	<100
		gr2 - 14	<100	<100	<100	<100	12986	<100	<100	<100	<100	<100
		gr2 - 15	<100	<100	<100	<100	2687	<100	<100	<100	<100	<100
		gr2 - 16	136	<100	<100	<100	8284	492	<100	<100	<100	<100
		gr2 - 17	119	<100	<100	<100	3231	<100	<100	<100	<100	<100
		gr2 - 18	267	<100	<100	<100	15153	<100	<100	<100	<100	<100
		gr2 - 19	114	<100	306	<100	2233	<100	<100	<100	<100	<100
		gr2 - 20	115	205	308	<100	6631	<100	<100	<100	<100	<100
		gr2 - 21	306	<100	235	<100	3322	<100	<100	<100	<100	<100
		gr2 - 22	111	<100	233	<100	5979	<100	<100	<100	<100	<100
		gr2 - 23	<100	<100	<100	<100	1391	<100	<100	<100	<100	<100
		gr2 - 24	207	<100	<100	<100	2719	<100	<100	<100	<100	<100
Rf-1 S	Standard	gr3 - 25	<100	<100	<100	<100	<100	<100	<100	<100	<100	<100
		gr3 - 26	240	<100	176	<100	589	<100	<100	<100	<100	<100
		gr3 - 27	<100	<100	<100	<100	267	<100	<100	<100	<100	<100
		gr3 - 28	<100	<100	<100	<100	<100	<100	<100	<100	<100	<100
		gr3 - 29	<100	<100	<100	<100	<100	<100	<100	<100	<100	<100
		gr3 - 30	<100	<100	<100	<100	<100	<100	<100	<100	<100	<100
		gr3 - 31	<100	<100	<100	<100	<100	<100	<100	<100	<100	<100
		gr3 - 32	<100	<100	<100	<100	<100	<100	<100	<100	<100	<100
		gr3 - 33	<100	<100	<100	<100	<100	<100	<100	<100	<100	<100
		gr3 - 34	<100	<100	<100	<100	436	106	<100	<100	<100	<100
		gr3 - 35	<100	<100	<100	<100	<100	<100	<100	<100	<100	<100
		gr3 - 36	<100	<100	<100	<100	<100	<100	<100	<100	<100	<100
Khosta-2 S	Standard	gr4 - 37	<100	<100	<100	<100	<100	<100	<100	<100	<100	<100
		gr4 - 38	<100	<100	<100	<100	<100	<100	<100	<100	<100	<100
		gr4 - 39	<100	<100	<100	<100	<100	<100	<100	<100	<100	<100
		gr4 - 40	143	<100	<100	<100	<100	<100	<100	<100	<100	<100
		gr4 - 41	<100	<100	<100	<100	<100	<100	<100	<100	<100	<100
		gr4 - 42	<100	<100	<100	<100	<100	<100	<100	<100	<100	<100
		gr4 - 43	<100	<100	<100	<100	<100	<100	<100	<100	<100	<100
		gr4 - 44	<100	<100	<100	<100	<100	<100	<100	<100	<100	<100
		gr4 - 45	<100	<100	<100	<100	<100	<100	<100	<100	<100	<100
		gr4 - 46	<100	<100	<100	<100	<100	<100	<100	<100	<100	<100
		gr4 - 47	<100	<100	<100	<100	<100	<100	<100	<100	<100	<100
		gr4 - 48	<100	<100	<100	<100	<100	<100	<100	<100	<100	<100
Mosaic	Standard	gr5 - 49	578	5852	2703	<100	939	<100	<100	<100	<100	<100
		gr5 - 50	2535	11852	17345	<100	9426	127	110	<100	<100	<100
		gr5 - 51	257	2339	1853	<100	1210	<100	<100	<100	<100	<100
		gr5 - 52	414	2367	7303	131	1497	<100	<100	<100	<100	<100
		gr5 - 53	<100	18386	915	<100	586	<100	<100	<100	<100	<100
		gr5 - 54	246	50780	1988	193	2277	<100	142	<100	<100	<100
		gr5 - 55	481	2886	1245	<100	1273	121	<100	<100	<100	<100
		gr5 - 56	268	13425	2897	<100	950	<100	<100	<100	<100	<100
		gr5 - 57	128	9898	1578	<100	247	<100	<100	<100	<100	<100
		gr5 - 58	280	9414	4990	<100	145	<100	<100	<100	<100	<100
		gr5 - 59	<100	6378	2629	<100	1298	<100	<100	<100	<100	<100
		gr5 - 60	<100	9449	2522	<100	1184	<100	<100	<100	<100	116
Cocktail	Standard	gr6 - 61	<100	11173	1882	<100	2197	110	<100	<100	<100	<100
		gr6 - 62	214	13373	6258	<100	6252	<100	<100	<100	<100	<100
		gr6 - 63	<100	6287	1111	<100	1018	<100	<100	<100	<100	<100
		gr6 - 64	<100	1982	1137	<100	225	<100	<100	<100	<100	<100
		gr6 - 65	<100	1564	218	<100	<100	<100	115	<100	<100	<100
		gr6 - 66	<100	22930	683	<100	355	<100	<100	<100	<100	<100
		gr6 - 67	665	10325	1342	<100	165	<100	<100	<100	<100	<100
		gr6 - 68	<100	7632	6869	<100	1175	<100	<100	<100	<100	<100
		gr6 - 69	251	8413	666	<100	657	<100	<100	<100	<100	<100
		gr6 - 70	<100	2057	5521	<100	3514	<100	<100	<100	<100	<100
		gr6 - 71	<100	829	2679	<100	852	<100	<100	<100	<100	<100
		gr6 - 72	273	5935	1540	<100	794	<100	<100	<100	<100	<100
Mosaic	Pre-vaccination	gr7 - 73	337	8956	13788	<100	3178	<100	<100	<100	<100	<100
		gr7 - 74	293	1586	1209	<100	3783	<100	<100	<100	<100	<100
		gr7 - 75	519	9869	12630	<100	2305	<100	<100	<100	<100	<100
		gr7 - 76	389	5174	3372	<100	4927	<100	<100	<100	<100	<100
		gr7 - 77	<100	34066	2066	<100	2045	<100	<100	<100	<100	<100
		gr7 - 78	139	8256	2275	<100	2173	<100	<100	<100	<100	<100
		gr7 - 79	757	25057	22954	104	12345	<100	<100	<100	<100	<100
		gr7 - 80	1003	59364	5361	<100	2605	<100	<100	<100	<100	<100
		gr7 - 81	326	31817	4634	<100	3124	<100	<100	<100	<100	<100
		gr7 - 82	220	3126	1779	<100	2346	<100	<100	<100	<100	<100
		gr7 - 83	203	5193	5149	<100	4823	<100	<100	<100	<100	<100
		gr7 - 84	1128	4093	1972	<100	2047	<100	<100	<100	<100	<100
Cocktail	Pre-vaccination	gr8 - 85	1153	32400	3050	<100	2946	<100	<100	<100	<100	<100
		gr8 - 86	642	3148	2163	<100	2189	<100	<100	<100	<100	<100
		gr8 - 87	<100	5275	2518	<100	318	<100	<100	<100	<100	<100
		gr8 - 88	916	4096	8767	<100	14467	<100	<100	<100	<100	<100
		gr8 - 89	<100	876	458	<100	237	<100	<100	<100	<100	<100
		gr8 - 90	1116	13626	6467	<100	3879	<100	<100	<100	<100	<100
		gr8 - 91	358	7662	2057	<100	1177	<100	<100	<100	<100	<100
		gr8 - 92	<100	<100	<100	<100	<100	<100	<100	<100	<100	<100
		gr8 - 93	318	10206	1901	<100	986	<100	<100	<100	<100	<100
		gr8 - 94	262	10215	8174	<100	2018	<100	<100	<100	<100	<100
		gr8 - 95	541	7452	4330	<100	421	<100	<100	<100	<100	<100
		gr8 - 96	734	17145	12177	<100	11181	<100	<100	<100	<100	<100
Mosaic	Pre-vaccination with extended protocol	gr9 - 97	5735	9838	15117	325	16932	1587	<100	<100	<100	<100
		gr9 - 98	893	14221	5873	<100	1971	102	<100	<100	<100	178
		gr9 - 99	2930	16848	17346	105	16792	538	<100	<100	<100	150
		gr9 - <100	368	9481	2107	103	7567	773	<100	<100	<100	347
		gr9 - 101	2493	3558	5499	109	4516	327	<100	<100	<100	175
		gr9 - 102	<100	356	<100	<100	<100	<100	<100	<100	<100	110
		gr9 - 103	1862	3269	4836	<100	1290	122	<100	<100	<100	<100
		gr9 - 104	2429	2525	11769	<100	8582	310	<100	<100	<100	<100
		gr9 - 105	1072	17207	11824	299	5399	316	<100	<100	<100	162
		gr9 - 106	2760	5805	13599	339	10037	500	<100	<100	<100	<100
		gr9 - 107	1417	10937	14162	175	18993	157	<100	<100	<100	<100
		gr9 - 108	212	4505	3510	<100	2994	121	<100	<100	<100	<100
Cocktail	Pre-vaccination with extended protocol	gr10 - 109	3258	14243	20012	<100	10866	385	<100	<100	<100	<100
		gr10 - 110	1017	7658	5504	160	7825	185	<100	<100	<100	<100
		gr10 - 111	<100	1486	609	<100	612	111	<100	<100	<100	<100
		gr10 - 112	774	2108	4224	<100	9906	167	<100	<100	<100	159
		gr10 - 113	406	16213	7624	<100	9932	458	<100	<100	<100	<100
		gr10 - 114	497	9853	5663	<100	4274	119	<100	<100	<100	<100
		gr10 - 115	1099	89608	18007	<100	5520	307	<100	<100	<100	<100
		gr10 - 116	<100	7588	2146	<100	1011	<100	<100	<100	<100	<100
		gr10 - 117	398	6199	4342	<100	13269	213	<100	<100	<100	101
		gr10 - 118	195	7511	5992	<100	2923	111	<100	<100	<100	<100
		gr10 - 119	904	3661	5371	<100	3146	100	<100	<100	<100	<100
		gr10 - 120	469	5767	5976	<100	983	<100	<100	<100	<100	<100

	**ID50**
	<100
	101 - 300
	301 - 3,000
	3,001 - 30,000
	>30,001
